# Critical analysis of ginkgo preparations: comparison of approved drugs and dietary supplements marketed in Germany

**DOI:** 10.1007/s00210-023-02602-6

**Published:** 2023-07-20

**Authors:** Milan Trabert, Roland Seifert

**Affiliations:** Institute of Pharmacology, Hannover Medical School, Carl-Neuberg-Str. 1, D-30625 Hanover, Germany

**Keywords:** Ginkgo biloba extract, GBE, EGb761, Alzheimer's dementia, Dietary supplements

## Abstract

Demographic change is taking place in the population of western industrialized countries, and the population is aging constantly. As a result, the mortality rate of patients due to dementia is rising steadily. To counteract this, the relevance of neuroprotective agents is increasing. Preparations from the medicinal tree species Ginkgo biloba (“gingko”) are becoming increasingly popular. In this study, 63 ginkgo preparations marketed in Germany were analyzed. The following data were collected from the package inserts of the preparations: Country of manufacture, approval as a drug, compliance to target values of flavone glycosides, compliance to target values of terpene lactones, compliance to target values of ginkgolic acids, dosage per unit in milligrams (mg), duration of use, interactions with other drugs, contraindications, adverse effects and daily defined dose costs. In the next step, these data were compared in the following form: Total preparations versus preparations with drug approval versus dietary supplements. Almost without exception, the results indicate a pharmaceutical reliability of the preparations with drug approval and a dubious reliability of the preparations marketed as dietary supplements. Thus, ginkgo preparations marketed as dietary supplements appear to have an economic rather than a medical focus. We discuss the evidence of efficacy, and other criteria mentioned above, to evaluate the adequacy of the costs for the statutory health insurance that pay for preparations with drug approval in Germany. From the analysis of our results it is very doubtful that ginkgo biloba extract preparations of the food industry have any health benefit. It must be evaluated whether prohibition of selling ginkgo biloba extract as a dietary supplement is an option.

## Introduction

Ginkgo biloba is one of the oldest medicinal plants. Its use as a medicinal plant date back to 1505 AD. The first uses of ginkgo can be traced back to China 5000 years ago (Xie et al. 2022). The ginkgo leaf extract was developed in Germany in 1965 for pharmaceutical purposes. The first commercially available ginkgo extract was approved for human use in France in 1974 under the name EGb761 (Isah 2015). In Germany, the Federal Joint Committee (GBA) commissioned the Institute for Quality and Efficiency in Health Care (IQWiG) in 2008 to assess the benefits of various drugs approved for the treatment of Alzheimer’s disease, which included preparations containing ginkgo.

Their plausibility was determined in the final report of the IQWiG in 2008 (https://www.iqwig.de/download/a0519b_abschlussbericht_ginkgohaltige_praeparate_bei_alzheimer_demenz.pdf, accessed 14 April 2023). According to IQWiG, a daily dose of 240 mg GBE provides a benefit for reaching the therapy goal “activities for daily living“. Furthermore, there are indications that ginkgo-containing preparations may contribute to the therapy goals “cognitive abilities“, “general psychopathological symptoms“ and “quality of life of (caring) relatives“. In 2011, the GBA passed a resolution to change the drug directive (AM-RL): Exhibit 1 (OTC Overview) No. 20 Ginkgo biloba leaf extract. Here, "Ginkgo biloba leaf extract (acetone-water extract, standardized) only for the treatment of dementia" was supplemented after the word "standardized" by: “240 mg daily dose”. (https://www.g-ba.de/downloads/40-268-1638/2011-04-14_AM-RL-OTC_Ginkgo_ZD.pdf, accessed 6 March 2023).

Dementia-related neurodegeneration is the seventh most frequent cause of death worldwide (https://www.who.int/news-room/fact-sheets/detail/dementia, accessed 2 October 2022). Since there are no effective drugs for Alzheimer's disease (AD) treatment currently (Hampel et al. 2020), increased attention is also being paid to alternative therapies (Bhattacharya et al. 2021) including Ginkgo biloba extract (GBE). GBE is one of the most widely used herbal remedies for dementia (DeFeudis and Drieu 2000). The combination of drugs and GBE is hoped to provide a new/more effective treatment option for AD (Chen et al. 2021). However, previous studies regarding the efficacy of GBE on AD show high heterogeneity (Yuan et al. 2017). A classic randomized trial showed no efficacy of ginkgo in the prevention of dementia (Dekosky, 2008), and the results of meta-analyses are controversial (Charemboon and Jaisin, 2015; Savaskan et al., 2018). The drug prescription report (AVR) for Germany has a very critical perspective on the use of GBE because of the lack of convincing clinical evidence and does not recommend prescription at the expense of the statutory health insurance (GKV) (Seifert and Petri 2021). Despite the lack of convincing clinical evidence, prescription of GBE in Germany is very popular.

This unsatisfying situation motivated us to investigate the characteristics of ginkgo preparations marketed in Germany. In this study, we analyzed GBE preparations with drug approval and approval as dietary supplements. So far, there is a lack of studies in this regard, although the dietary supplement preparations of the food industry enjoy a particularly wide reach in the population (https://marketresearch.biz/report/ginkgo-biloba-extract-market/, accessed 2 October 2022). The data collected were analyzed for the totality of preparations, preparations with drug approval as well as preparations with approval as dietary supplement.

## Materials and methods

### Analysis of the individual preparations

The package inserts of the products were used for the analysis of the individual preparations, which are available for download on the following online pharmacies (as of 23.09.2022):

- https://www.shop-apotheke.com/
- https://shop.apotal.de/
- https://www.medikamente-per-klick.de/

Each retrieval date of the leaflet used for data collection was documented in tabular form. The following criteria were recorded: Screenshot of the preparation, packaging presentation/design, product name, Pharmaceutical Central Number (PZN) / European Article Number (EAN), brand, manufacturer, country of manufacture, presentation, pack size, recommended retail price (RRP) / pharmacy retail price (AVP), cost per unit, dosage per unit in milligrams (mg), DDD cost (defined daily dose), duration of use, active ingredients, flavone glycosides (nominal value according to the German Pharmacopoeia/DAB and the European Pharmacopoeia/Pharmacopoeia Europaea/Ph.Eur. 22-27%) per unit, terpene lactones (nominal value/DAB and Ph.Eur. 5-7% of which 2.8-3.4% are ginkgolides A, B, C and 2.6-3.2% is bilobalide) per unit, ginkgolic acids (nominal value/DAB and Ph.Eur. <5 parts per million/ppm) per unit, other ingredients, interaction with other drugs, contraindications, adverse effects, approval as a drug (pharmacy requirement), consumer-friendly indication of target values in percent, website, website retrieval date.

## Statistical analysis of all preparations

Using the data collected, further analyses were performed. These were divided into: Total preparations, preparations with drug approval and dietary supplement products. These three categories were analyzed with regard to the following criteria: Compliance to target values of flavone glycosides, compliance to target values of terpene lactones, compliance to target values of ginkgolic acids, dosage per unit in milligrams, information regarding duration of application, interactions with other drugs, contraindications, adverse drug reactions, maximum DDD costs.

## Results

**Fig. 1:**
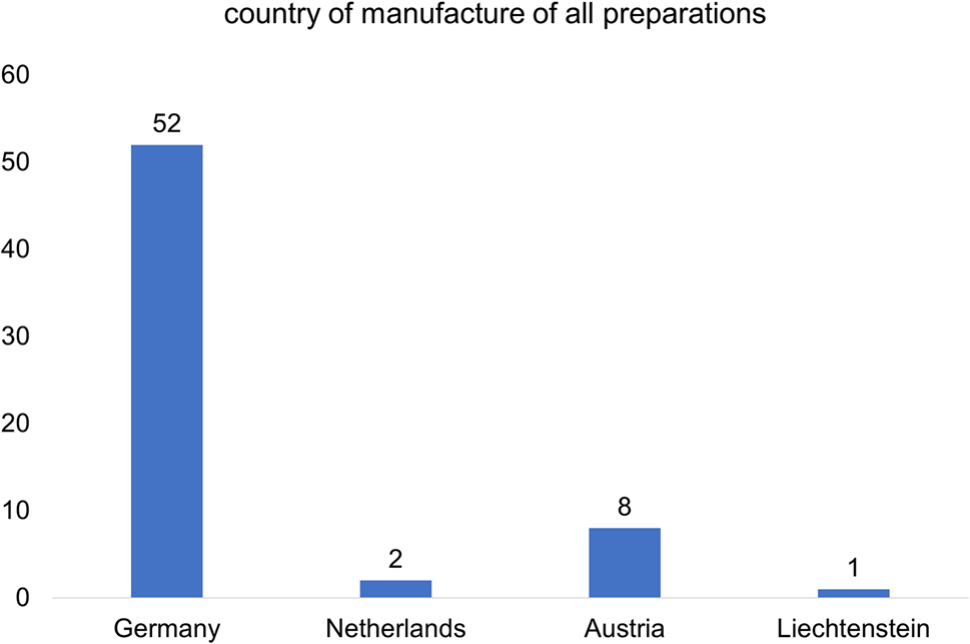
Analysis of the countries of manufacture of the total 63 preparations

Figure 1 shows the proportion of the total 63 preparations marketed in Germany in relation to their country of manufacture. The majority of the preparations (52) are manufactured in Germany. This is followed by Austria with 8 preparations. Two preparations are manufactured in the Netherlands, and one in Liechtenstein.

**Fig. 2:**
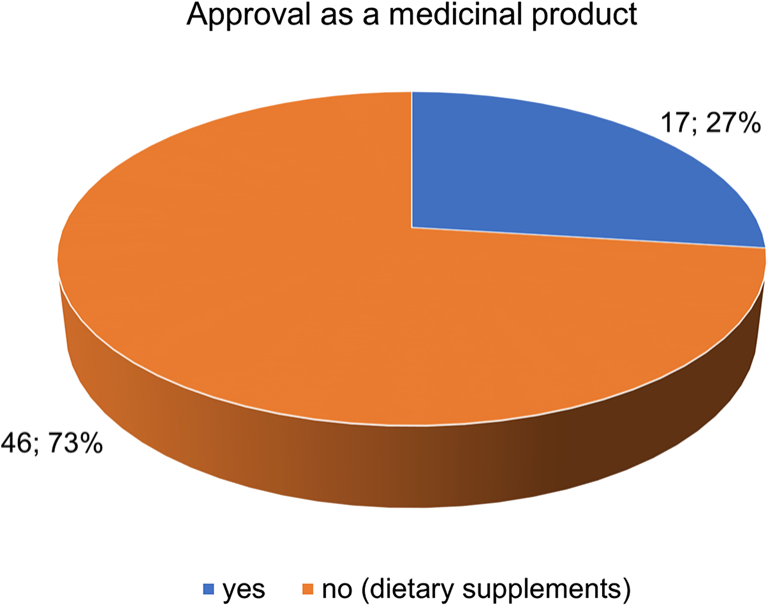
Comparison of approval as a drug/pharmacy and approval as a dietary supplement, of the total 63 preparations

Figure 2 compares the total of 63 preparations with regard to their approval as medicinal products (drugs) and approval as dietary supplements. The majority (46 preparations; 73%) are not approved as medicinal products but as dietary supplements. Seventeen preparations (27%) are approved as medicinal products and subject to pharmacy authorization.

**Fig. 3:**
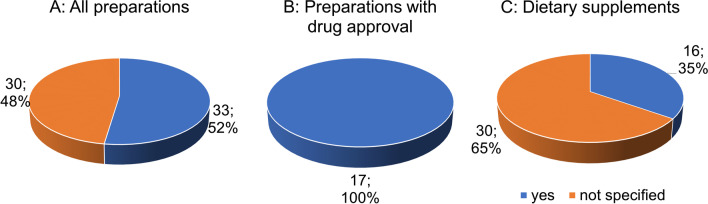
Analysis of the compliance to the target values of flavone glycosides

Figure 3 shows the compliance to the target values of flavone glycosides (target/DAB and Ph.Eur. 22-27%) per unit (Savaskan et al., 2018). Figure 3A represents how many of the total 63 preparations meet the required target values of flavone glycosides. A slight majority (33 preparations; 52%) meet the target values, whereas no information is provided for 30 preparations (48%). Figure 3B shows that all 17 preparations with drug approval meet the required target values for flavone glycosides. Figure 3C shows that the majority of products approved as dietary supplement provides no data (30 preparations, 65%). Compared to the total of 63 preparations (Fig. 3A), this proportions is 17% higher. Sixteen preparations (35%) meet the required target values.

**Fig. 4:**
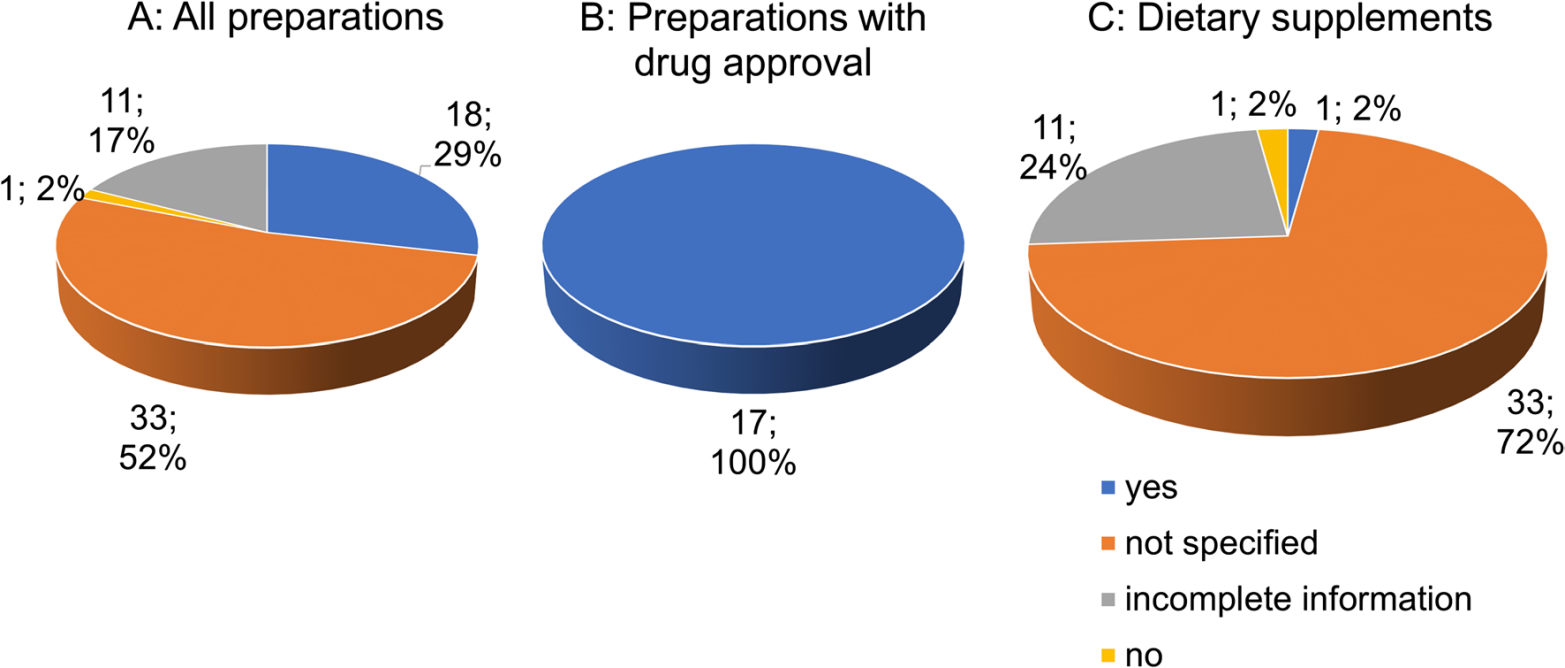
Analysis of the compliance to the target values of terpene lactones

Figure 4 shows the compliance to the target levels of terpene lactones (target/DAB and Ph.Eur. 5-7% of this 2.8-3.4% ginkgolides A, B, C and 2.6-3.2% bilobalide) per unit (Savaskan et al. 2018). Figure 4A represents how many of the total 63 preparations meet the required target levels of terpene lactones. Most products provide no data (33 preparations; 52%). All preparations with drug approval (29%) meet the required target values (Fig. 4B). Among dietary supplements, most preparations fail to provide the required information (Fig. 4C).

**Fig. 5:**
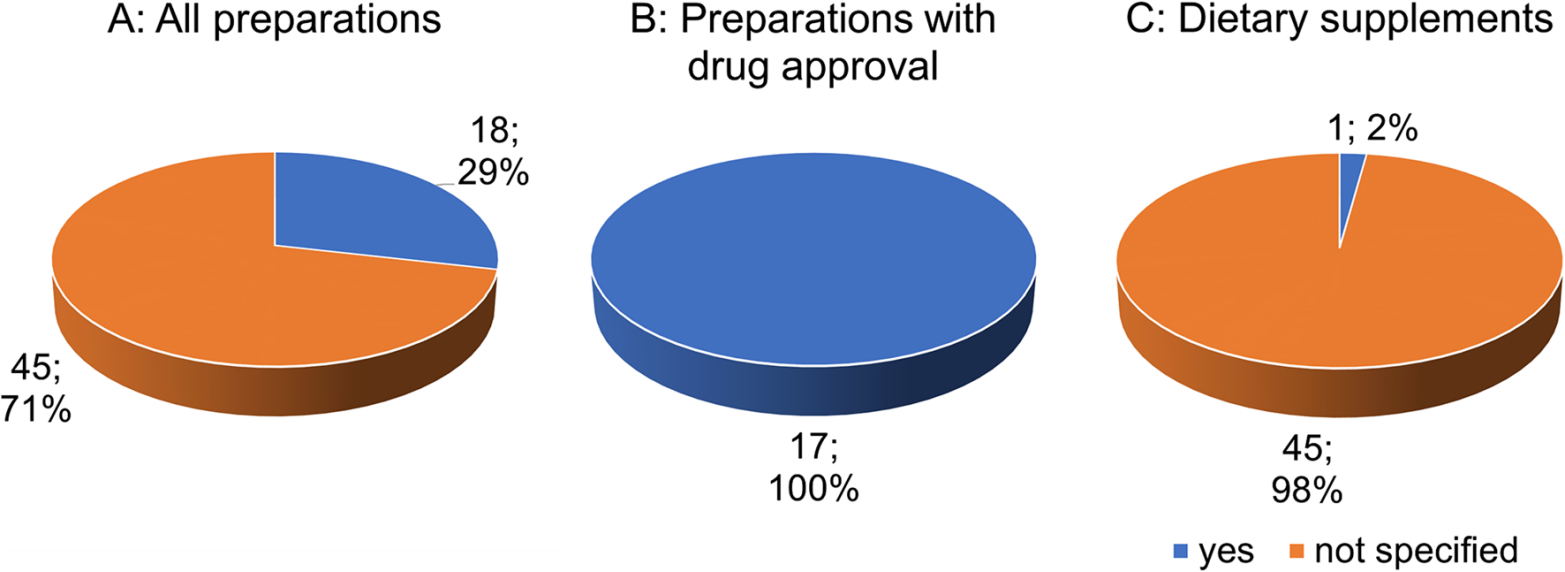
Analysis of the compliance to the target values of ginkgolic acids

Figure 5 shows the compliance to the maximum target values of ginkgolic acids (target value/DAB and Ph.Eur. <5 ppm) per unit According to the Commission E of the former German Federal Health Agency, the value of 5 ppm is defined as the maximum amount of ginkgolic acids per unit and must not be surpassed due to the toxicity of the acid (Mei et al. 2017) For the majority (45 preparations;/ 71%), no information is provided (Fig. 5A). In 18 preparations (29%), the required target values of ginkgolic acids are met. All 17 preparations with drug approval meet the required target values (Fig. 5B). Most dietary supplements (45 preparations; 98%) do not provide any information, and only one preparation (2%) meets the required target values (Fig. 5C).

**Fig. 6:**
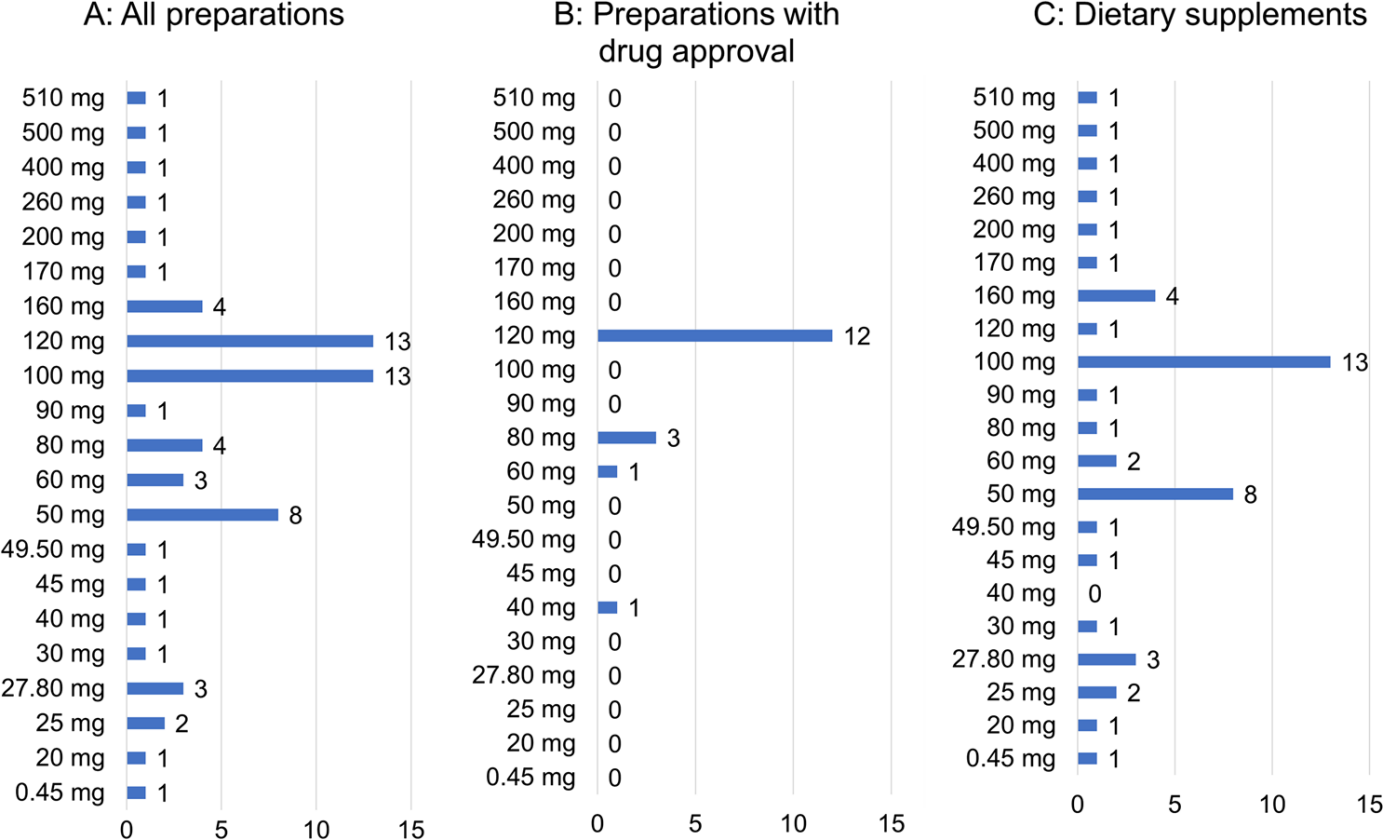
Analysis of dosage per unit in milligrams (mg)

Figure 6A shows the distribution of dosage per unit in milligrams (mg) of the total 63 preparations. The majority, 13 preparations (21%), contain 100 mg or 120 mg per unit, followed by 8 preparations (13%) with 50 mg per unit. Beyond this, there is a wide variation in dosage. Figure 6B shows the distribution of dosage per unit (mg) related to the 17 preparations with drug approval. The dosage is distributed in 4 ranges; 120 mg dominates with 12 preparations (70%), followed by 80 mg (3 preparations; 18%). Then, one preparation with 40 or 60 mg follows. Figure 6C shows the distribution of preparations without drug approval, with the spectrum of mg quantity being much more diverse. Here, 100 mg dominates with 13 preparations, followed by 50 mg with 8 preparations. The further distribution is rather heterogeneous.

**Fig. 7:**
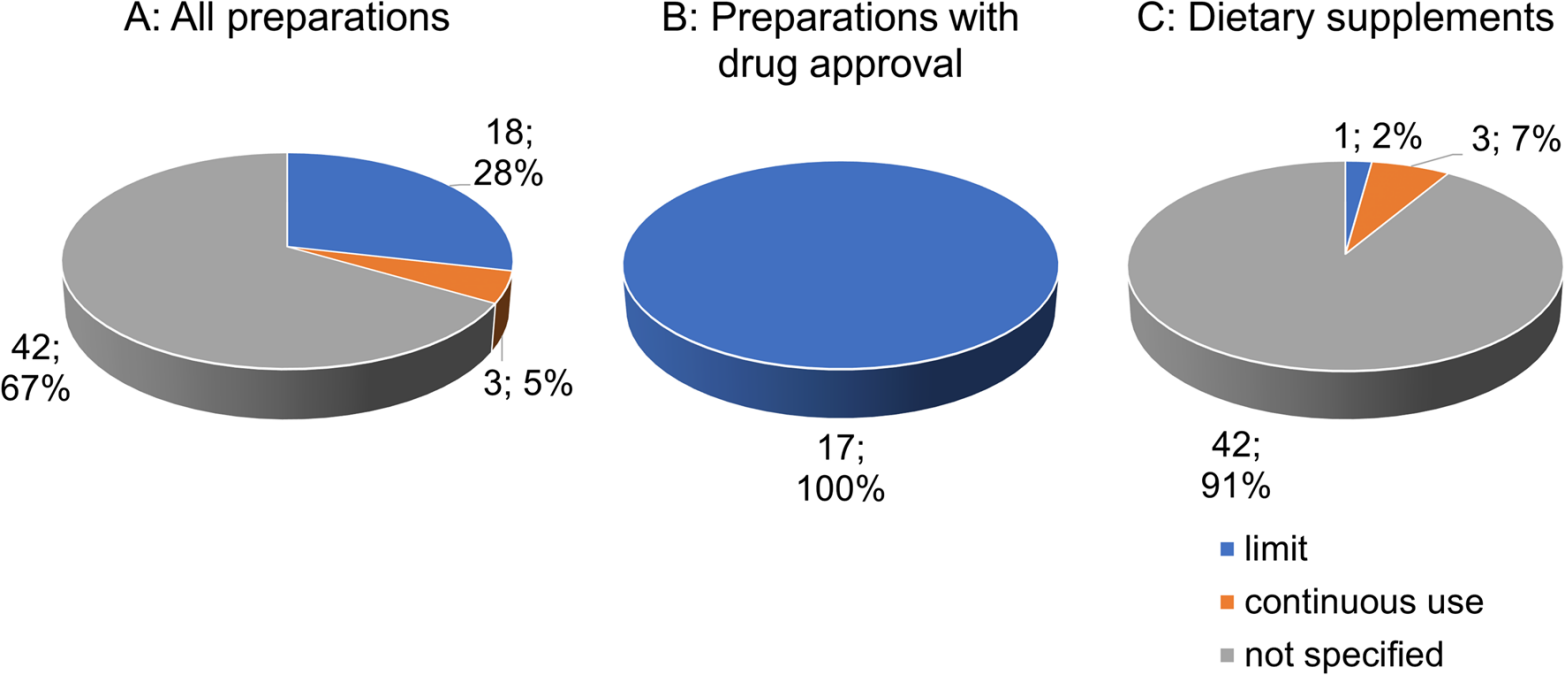
Analysis of information regarding the duration of use

Figure 7A shows the distribution of the 63 preparations in relation to the duration of use. In the majority (42 preparations; 67%) no information is given. Eighteen preparations (28%) give a limit for the duration of use. Three preparations (5%) recommend continuous use. Figure 7B shows that all 17 preparations with drug approval indicate a limit for the duration of intake. Figure 7C shows the distribution of the 46 preparations without drug approval for the duration of use. In most cases (42 preparations; 91%) no information is given. Three preparations (7%) advise continuous use and one preparation (2%) gives a limit.

**Fig. 8:**
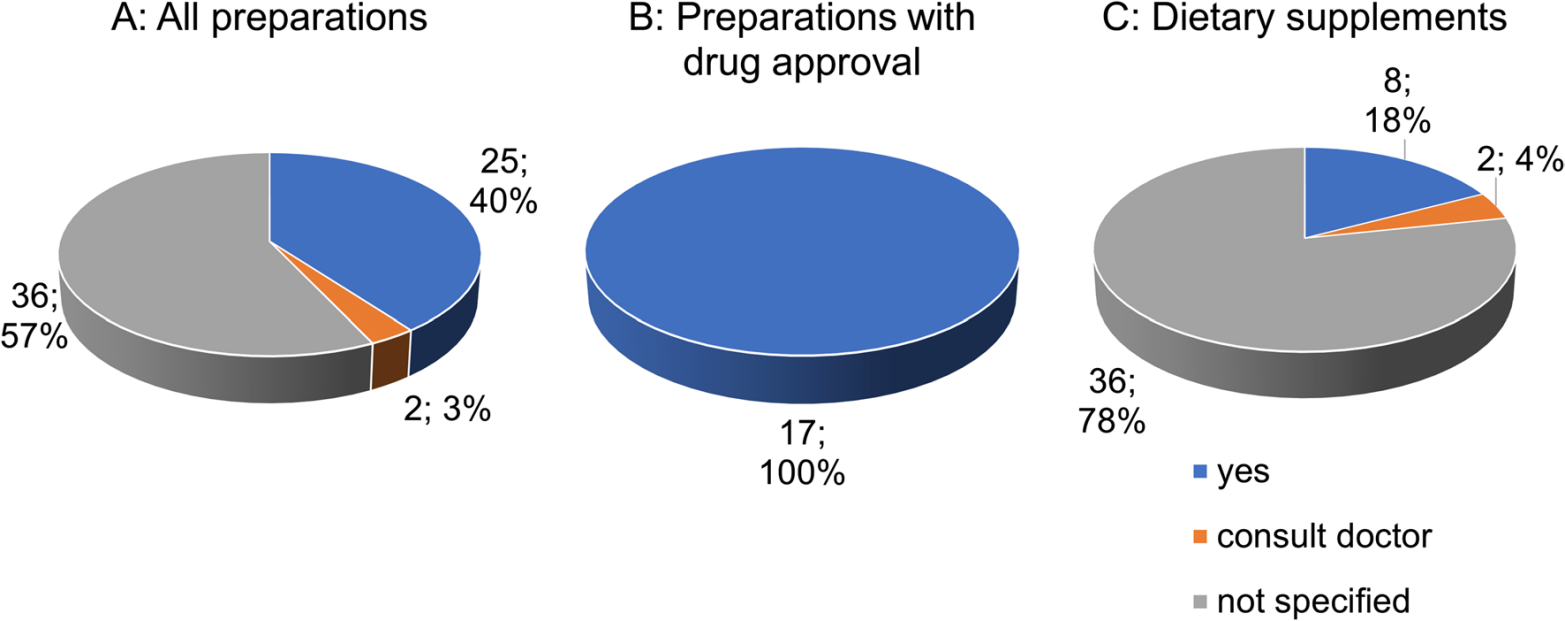
Analysis of the indication of drug-drug interactions

Figure 8A shows the distribution of information on interactions with other drugs among the 63 preparations. The majority (36 preparations; 57%) do not provide any information on this topic. Twentyfive preparations (40%) provide specific information on interactions with other drugs. Two preparations (3%) advise to consult a doctor in this regard. All 17 preparations with drug approval provide specific information on interactions with other drugs (Fig. 8B). The majority of dietary supplements (36 preparations; 78%) do not provide any information on interactions with other drugs. Specific information is provided for 8 preparations (18%). Consultation with a physician is advised for 2 preparations (4%).

**Fig. 9:**
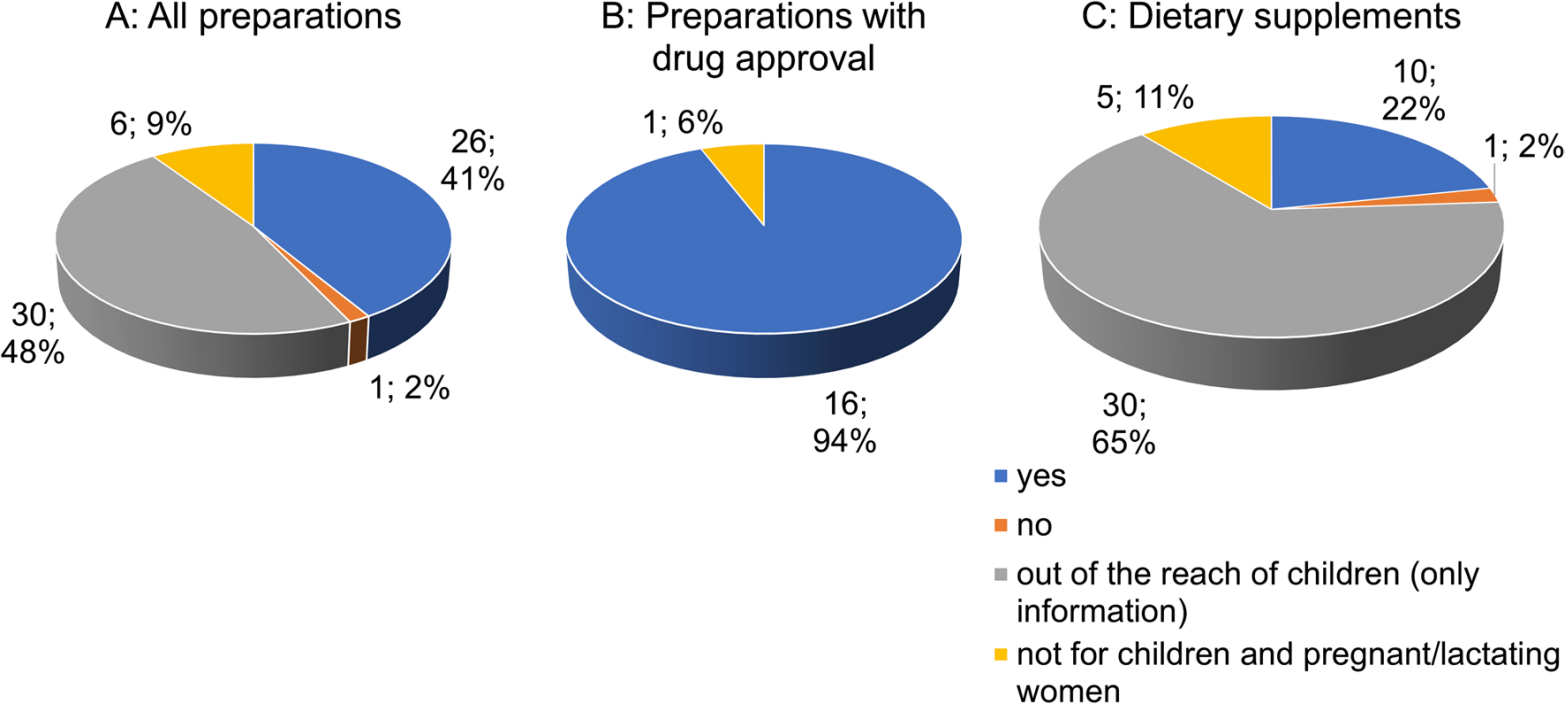
Analysis of the specification of contraindications

Figure 9A shows the distribution of contraindications in relation to the 63 preparations. For the majority (30 preparations; 48%), "keep out of reach of children" is the only indication given, followed by 26 preparations (41%), naming specific contraindications. Six preparations (9%) advise against use for children and during pregnancy/breastfeeding. One preparation (2%) does not specify any contraindications. The majority of preparations with drug approval (16 preparations; 94%) gives specific information on contraindications (Fig. 9B). One preparation (6%) advises against use for children and during pregnancy/breastfeeding. Most dietary supplements (30 preparations; 65%) indicate "keep out of reach of children" as the only indication. Ten preparations (22%) give specific information on contraindications. Five preparations (11%) advise against use for children and during pregnancy/breastfeeding and 1 preparation (2%) does not specify contraindications.

**Fig. 10:**
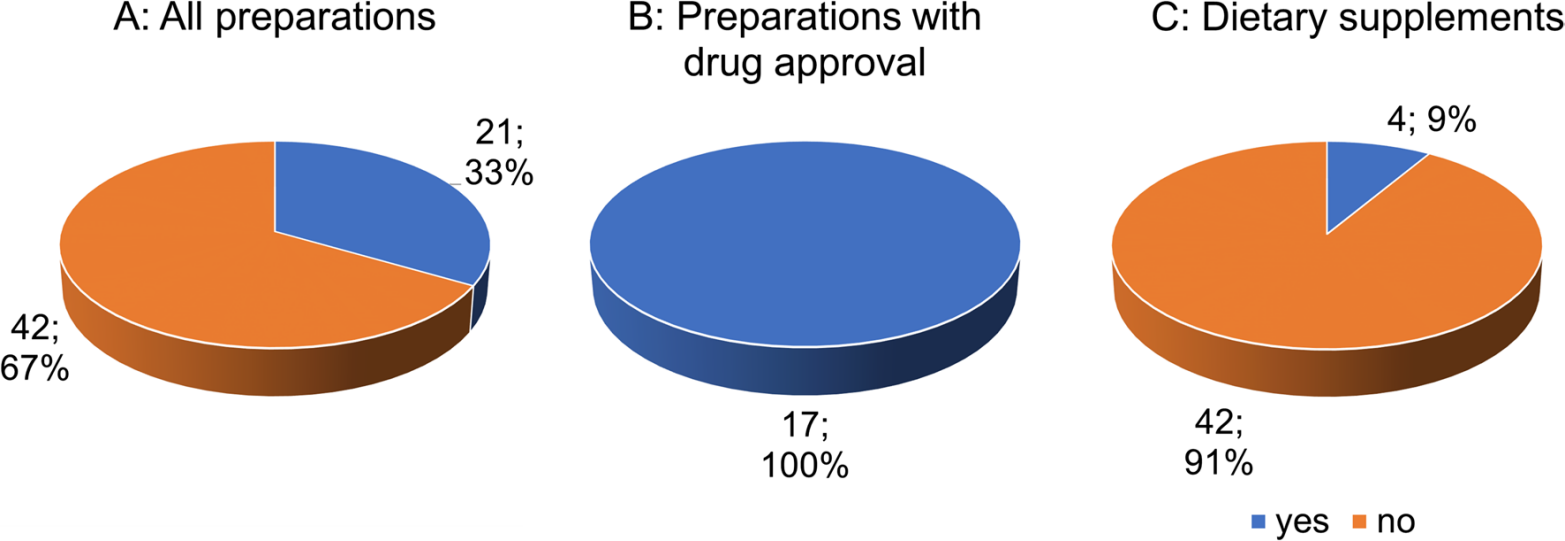
Analysis of the indication of adverse effects

Figure 10A shows the distribution of adverse effects in relation to the 63 preparations. For the majority (42 preparations; 67%), no information is given on adverse effects. Twenty-one preparations (33%) specifically state adverse effects. For all 17 approved drugs, concrete information regarding adverse effects is provided (Fig. 10B). Most dietary supplements (42 preparations; 91%) do not provide any statements regarding adverse effects (Fig. 10C).

**Fig. 11:**
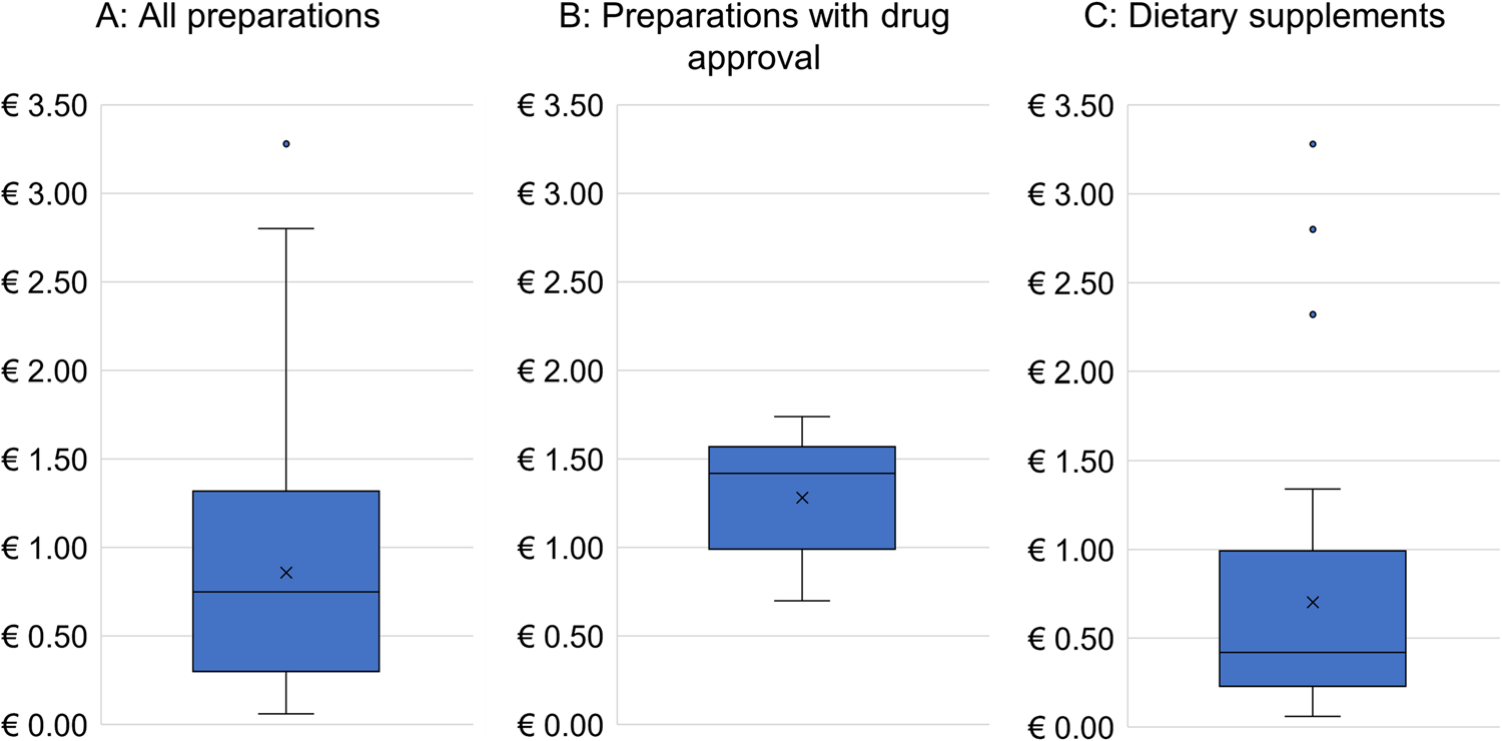
Analysis of maximum DDD costs. The box corresponds to the area containing the middle 50% of the data. It is bounded by the upper and lower quartiles. The line centered in the blue boxes mark the median values. The crosses represent the mean values. The upper whiskers mark the maximum and the lower whiskers mark the minimum values. The dots outside the boxes denote outsiders

Figure 11A presents the maximum DDD costs of the total 63 preparations in a box plot diagram. The median is € 0.75; the mean is € 0.86 Fig. 11B shows the 17 compounds with drug approval in terms of maximum DDD costs. The median of the preparations with drug approval was higher (€ 1.42) than the median of the total 63 preparations. There are no outliers. The median of the dietary supplements was lower (€ 0.42) than the median of the preparations with drug approval. There are 3 high outliers to the top.

## Discussion

### Analysis of the countries of manufacture of the preparations

Most GBE preparations marketed in Germany are produced in Germany (Fig. 1). In the 1970s, Dr. Schwabe (Karlsruhe, Germany) developed GBE/EGb 761 (Drieu and Jaggy 2000) which has also been available throughout Europe since the 1990s. In the USA, EGb 761 is not approved for use in pharmacies by the US Food and Drug Administration (FDA). Nature's Way (USA), however, uses the EGb 761 formula for dietary supplements (Li et al. 2022). While GBE is approved as a drug and dietary supplement in Europe, it is only approved as a dietary supplement in the USA. This highlights a substantial cultural difference in drug approval and use.

### Comparison of marketing as a drug and as a dietary supplement

In the German Drug Prescription Report (AVR) 2021, GBE is listed in Table 28.2 on p. 566 (Seifert and Petri 2021). Although there is no proven benefit, GBE is prescribed with increasing tendency. According to German S3 guideline Dementias, GBE can be considered for mild to moderate Alzheimer's dementia (Seifert and Petri 2021). In addition to ginkgo preparations which are approved as drugss, dietary supplements that are ginkgo-based are widely used worldwide (Nguyen and Alzahrani 2022). Dietary supplements do not require the extensive and expensive approval by drug agencies. Likewise, there may be very substantial discrepancies between labeled and actual ingredients and their amounts (Nguyen and Alzahrani 2022). From the standpoint of safety and efficacy of dietary supplements, recommending such dietary supplements should be done judiciously by treating physicians (Nguyen and Alzahrani 2022). In Germany, legal disputes have repeatedly arisen as to when Ginkgo preparations must be approved as drugs or as dietary supplements (https://www.aerztezeitung.de/Politik/Zulassung-als-Arzneimittel-nur-notwendig-wenn-Risiken-bestehen-404035.html, accessed 2 October 2022).

Of the total of 63 preparations, 46 (73%) are approved as dietary supplements. Seventeen preparations (27%) are approved as drugs and are available only in pharmacies (Fig. 2). The fact that most ginkgo preparations are only approved as dietary supplements could be due to the fact that they have to fulfill fewer requirements, which at the same time must be regarded as a reason for caution. Furthermore, financial hurdles of the drug approval process could be relevant for the manufacturers. Another reason for the high proportion of GBE preparations approved as dietary supplements could be the high market potential. GBE products are among the most commercialized dietary supplements worldwide (Grass-Kapanke et al. 2011). These products should therefore be carefully analyzed for their ingredients and claims regarding their use.

### Analysis of the compliance to the target values

EGb 761 is an extract of ginkgo biloba leaves. It contains 22.0%-27.0% ginkgo flavonoids, 5.0%-7.0% terpene lactones (consisting of 2.8%-3.4% ginkgolides A, B, C and 2.6%-3.2% bilobalide). Also included are less than 5ppm ginkgolic acids, and the extractant is acetone 60% (w/w) (Savaskan et al. 2018).

The main active ingredients of EGb 761 are flavone glycosides. These are believed to be involved in inhibiting inflammatory cytokines (Zhang et al. 2018). Furthermore, flavone glycosides have antioxidant activities in cell studies in which they scavenge free radicals, superoxide anions, and nitric oxide (Zuo et al. 2017). Flavonoids are used to prevent cardiovascular diseases and lower blood lipids and cholesterol. Therefore, they may serve to prevent vascular symptoms in the context of AD (Gohil et al. 2000). Terpene lactones constitute the second major component among the active compounds (Shi et al. 2009). Bilobalide and ginkgolides A-C belong to the terpenoids (Xie et al. 2022). In addition, ginkgolide B may attenuate neurotoxicity induced by ß-amyloid (Shi et al. 2009). Ginkgolides A, B, and C are the primary active ingredients in GBE related to inhibition of platelet aggregation (Xu et al., 2017). Furthermore, terpene lactones exhibit mitochondria-protective properties (Abdel-Kader et al. 2007). However, as the ginkgolic acids are the potentially toxic components in GBE, the target value of 5 ppm per unit may not be surpassed (Qian et al. 2017).

Although the toxicity of ginkgolic acids has not yet been precisely clarified, oral administration of ginkgolic acids to male rats at both high doses (900 mg/kg) and low doses (100 mg/kg) indicates hepatotoxicity and nephrotoxicity. A more detailed clarification of potential toxicity is urgently needed (Qian et al. 2017). In our analysis, we show that many GBE products deviate from the EGb 761 formula. It also became apparent that GBE preparations from the food industry frequently meet the value of flavone glycosides as the only target value. The reason for this may be that those GBE products are often adulterated with inexpensive plant materials containing individual flavone glycosides or flavonols (Harnly et al. 2012). This would reduce the potential efficacy of dietary supplements. Among dietary supplements, however, the occurrence of toxic effects due to ginkgolic acids is also possible. Most dietary supplements do not specify the concentration of ginkgolic acids. Ginkgolic acids are associated with a mutagenic (Westendorf and Regan 2000), cytotoxic (Hecker et al. 2002) and allergenic (Koch et al. 2000) activity. Studies conducted by the US National Toxicology Program (NTP) show that GBE has evidence of carcinogenic activity in rats and mice (Mei et al. 2017). GBE has been classified by the International Agency for Research on Cancer in group 2B as possibly carcinogenic to humans (Mei et al. 2017). Thus, exceeding the standard values ​​for ginkgolic acid could not only result in a lack of a positive effect, but also a toxic effect. In fact, some ginkgo dietary supplements contain high concentrations of ginkgolic acids (Mei et al. 2017).

### Unit dosage analysis in milligrams (mg)

Clinical studies to date indicate that long-term administration of GBE up to 240 mg/day is safe (Herrschaft et al. 2012). Furthermore, a dose of 240 mg/day for standardized GBE is recommended as most effective according to dementia treatment guidelines (Ihl et al. 2011). This dose represents the maximum recommended dose for GBE (Unger 2013). In contrast, a dose of 120 mg/day does not appear to ensure a response (Hashiguchi et al. 2015). However, many products have a lower GBE content. This requires multiple units per day for most preparations, which in turn increases the daily costs for the consumer and possibly higher costs for the statutory health insurance. Furthermore, large outliers upward should be regarded as dubious/unsafe. High doses could lead to adverse effects, since according to guidelines safe intake is only given up to 240 mg/day. In addition, a higher mg quantity per unit could suggest a potentially higher effect to the consumer, although this is not the case. The preparations that are dosed under 240 mg could lead to increased costs due to multiple intake, while preparations that are dosed over 240 mg exceed the recommended maximum dose. Large deviations from a dosage unit of 120 mg in numerous dietary supplements could therefore reflect economic interests.

### Indication of the duration of use

In clinical AD studies, partial improvements in cognitive abilities may become evident after 6 months of intake, but not with short-term intake of less than 6 months. This may be because GBE cannot cross the blood-brain barrier with high efficiency (Di Martino et al. 2017). Furthermore, clinical studies showed that in addition to the dose of 240 mg/day, long-term intake (6 months) is also safe. A dose of 240 mg/day administered over 22 weeks appeared to be more effective than taking a placebo in a particular study (Hashiguchi et al. 2015). Accordingly, a high dose (240 mg/day), as well as long-term administration (over 24 weeks) seem to be crucial for a potentially neuroprotective effect in early stages of AD (Xie et al. 2022).

Not specifying duration of use, or advice on continued use could both be for profit reasons. Consumers could consume these preparations for their lifetime and thus generate constant income for the manufacturers. Furthermore, consumers have no benchmark in this way to which they can orientate themselves. 

### Indication of interactions with other drugs

Interactions of GBE with cyclooxygenase inhibitors (COX inhibitors; traditionally referred to as nonsteroidal anti-inflammatory drugs (NSAIDs)), antiplatelet agents, anticoagulant therapy, and acetylsalicylic acid are possible (Rosenblatt and Mindel 1997). Administration of GBE and warfarin correlates with an increased risk of bleeding (Stoddard et al. 2015). GBE has properties of a monoamine oxidase inhibitor and thus can induce serotonin syndrome in patients taking antidepressants (White et al. 1996).

The failure to state drug interactions in most GBE dietary supplements thus represents a potential health hazard for the consumer. One reason for not specifying the interactions could be to reach a broader customer base. For a professional and harmless application, it is urgently required that the drug interactions are specifically listed even in dietary supplements.

### Specification of contraindications

The use of GBE in patients with blood clotting disorders is a potential contraindication (Rosenblatt and Mindel 1997). Since there is insufficient evidence of perioperative risk associated with GBE use, GBE should be discontinued at least 36 h before surgery (Ang-Lee et al. 2001). Also with regard to pregnancy/breastfeeding and infants, there is a lack of evidence for a risk-free intake of GBE; therefore intake is not recommended in these cases either (Anonymous 2006). Another contraindication is given for epilepsy patients. Ginkgotoxin, which is present in ginkgo seeds and ginkgo leaves, could lower the seizure threshold (Jang et al. 2015). GBE has effects on blood glucose, so increased monitoring of blood glucose levels is recommended in diabetic patients (Xin et al. 2014). However, there is a paucity of information on contraindications in dietary supplements.

### Adverse effects statement

Taking GBE may cause side effects such as headache, palpitations, constipation, allergic skin reactions, and gastrointestinal disturbances (Anonymous 2006). There are several case reports describing an association between GBE ingestion and bleeding events and severe intracranial hemorrhage (Kellermann and Kloft 2011). However, most dietary supplements do not provide any information on adverse effects.

### Maximum DDD (defined daily dose) cost analysis

The German Drug Prescription Report 2021 notes an increase in DDD net costs from 2019 to 2020 of 158.9% to € 0.64 of a specific ginkgo biloba product (Seifert and Petri 2021). This increase shows in a representative way the development of a greater context (Seifert and Petri 2021) and underlines the high market potential of GBE products.

Since for many preparations the prescribed dosage is based on symptoms, the maximum listed DDD costs of the preparations were compared as a reference value. In contrast to dietary supplements, the pricing of all prescription drugs in Germany is regulated by the Drug Price Ordinance (AMPreisV). This difference in regulation could explain the wide range of variation among dietary supplements, which are subject to free competition. However, it seems questionable why there are also deviations in the DDD costs among the preparations with drug approval, as some of the ingredients are the same. Since the health insurance companies bear the main costs for prescription drugs, discount agreements between the health insurance companies and the drug manufacturers could be the reason for possible price fluctuations. Nevertheless, these price fluctuations are much lower than for dietary supplements due to the stricter regularization. The stricter regulations could also ensure a fairer price for the preparations approved as medicinal products. Some dietary supplements exhibit exceptionally high DDD costs, well surpassing the most expensive drug. The “fantasy” DDD costs of some dietary supplements highlight the large market potential of the products and the medical/nutritional need from the consumer side.

### Limitations of this study and some future studies

The analyses and results of this study are based exclusively on information from the publicly available package inserts of the respective manufacturers of the preparations. Only information that could be found in the package inserts was included in the study. If the information provided in the package insert had been incomplete, this could have led to a distortion of the results. Unfortunately, we had no access to data on sales numbers of ginkgo dietary supplements. This would be important to estimate the overall size of the problems associated with these preparations.

Moreover, all company headquarters are based in European countries. For future studies, a comparison with US-based manufacturers would be interesting, especially with regard to the preparations without a drug approval. Since the Dietary Supplement Health and Education Act (DSHEA) was passed by the US Congress in 1994, NEGs have been placed in a special food category in the US (Mei et al. 2017). Thus, NEGs can be marketed without US Food and Drug Administration (FDA) approval of efficacy and safety (Mei et al. 2017).

In a study, 27 Ginkgo biloba leaf extract products from health food stores and supermarkets were compared for compliance with standard norms. Seventeen products deviate from the norm, with regard to ginkgolic acids even up to 90,000 ppm, surpassing allowed maximum values applicable to Germany by almost 20,000-fold (Mei et al. 2017). It is important to determine the concentration of ginkgolic acids in the preparations analyzed herein. In this way, the risk of toxicity of ginkgo dietary supplements marketed in Germany could be made apparent.

### Conclusions

Many ginkgo preparations that are marketed as dietary supplements do not meet the target values ​​for EGb 761. This means that there is not only no supposedly positive effect, but that there could even be toxic effects due to ginkgolic acids. In contrast, preparations with drug approvals meet the required target values. In addition, the wide range of variation in DDD costs and dosage units in dietary supplements must be questioned. To counteract the misleading of consumers, preparations with drug approval should be specifically labeled. The same applies to dietary supplements. For reasons of consumer protection, particularly because of the possible toxicity and carcinogenicity, it may even make more sense to take the dietary supplements from the market and only allow drugs that have undergone an approval process and clinical studies. In the case of dietary supplements, information is also often not or only insufficiently provided about the duration of use, drug interactions, contraindications and adverse effects, which constitutes a risk to the consumer. In general, it seems as if dietary supplement ginkgo preparations primarily pursue financial interests to satisfy the increasing consumer demand instead of acting in a health-oriented manner.

## Data Availability

All source data for this study are available upon reasonable request.

## Abbreviations

AD: Alzheimer´s disease
AMPreisV: Arzneimittelpreisverordnung (Drug price regulation)
AVP: Apothekenverkaufspreis (pharmacy retail price)
AVR: Arzneiverordnungsreport (Drug prescription report)
DAB: Deutsches Arzneibuch (German Pharmacopoeia)
DDD: Defined daily dose
DSHEA: Dietary Supplement Health and Education Act
EAN: European Article Number
FDA: US Food and Drug Administration
GBA: Gemeinsamer Bundesausschuss (Federal Joint Committee)
GBE: Ginkgo biloba extract
IQWiG: Institut für Qualität und Wirtschaftlichkeit im Gesundheitswesen (Institute for Quality and Efficiency in Health Care)
NO: Nitric oxide
NSAIDs: nonsteroidal anti-inflammatory drugs
NTP: National Toxicology Program
OTC: over-the-counter drug
Ph. Eur.: Pharmacopoeia Europaea (European Pharmacopoeia, Europäisches Arzneibuch)
PZN: Pharmazentralnummer (Pharmaceutical Central Number)
RRP: Recommended Retail Price (unverbindliche Preisempfehlung, UVP)
SHI: Statutory health insurance (Gesetzliche Krankenversicherung, GKV)
